# Dexmedetomidine dosage in critically ill patients undergoing intraoperative wake-up test: A randomized controlled trial

**DOI:** 10.1097/MD.0000000000028993

**Published:** 2022-03-11

**Authors:** Ting Yang, Muhammad Saqib Mudabbar, Tao Chen, Hong Jia, Qiang Fu, Bin Liu

**Affiliations:** a Department of Anesthesiology, West China Hospital, Sichuan University, #37 Guoxue Alley, Wuhou District, Chengdu, Sichuan Province, China; b Department of Anesthesiology, Chengdu Third People's Hospital Affiliated to Southwest Jiaotong University, Chengdu, Sichuan, China; c Department of Cardiovascular Medicine, West China Hospital, Sichuan University, Wuhou District, Chengdu, Sichuan Province, China; d Department of Rehabilitation, Affiliated Hospital of Southwest Medical University, Luzhou City, Sichuan Province, China.

**Keywords:** dexmedetomidine, different doses, intraoperative wake-up test, spinal orthopedic scoliosis correction surgery

## Abstract

**Objective:**

The aim of this study was to find the optimum dosage of dexmedetomidine in Spinal Orthopedic Scoliosis Correction Surgery when used in combination with propofol and remifentanil in American Society of Anesthesiologists (ASA) III patients with severe scoliosis undergoing intraoperative wake-up test.

**Materials and methods:**

We selected a total of 60 ASA III ≤40 years old patients who underwent Spinal Orthopedic Scoliosis Correction Surgery (SOSCS) and randomized them into groups A, B, and C. Group A was administered 0.2 μg/(kg·h) of dexmedetomidine, group B 0.3 μg/(kg·h), and group C 0.4 μg/(kg·h). The main parameters monitored were: wake-up time; wake-up quality; adverse effects that occur while the patient is awake; postoperative awareness of intraoperative wake-up test; heart rate (HR); mean arterial pressure (MAP); and oxygen saturation (SpO_2_). Values of these parameters were monitored at 7 timestamps separated by 5 minutes >30 minutes.

**Results:**

Group B had a higher MAP at 10 minutes before wake-up (*P* = .03) and at the moment of wake-up (*P* = .04) than group A. The Wake-up time of group A was 14.95 ± 7.42 minutes, group B was 14.7 ± 6.52 minutes, which was significantly shorter than that of group C 21.3 ± 10.02 minutes (*P* = .02). The wake-up quality was excellent. All other parameters had no significant statistical differences.

**Conclusion:**

Doses of 0.2 to 0.3 μg/(kg·h) have shorter wake-up time and fewer hemodynamic fluctuations compared to 0.4 μg/(kg·h).

## 1 Introduction

Severe scoliosis is defined as having a spinal curvature of Cobb's angle >45 degrees or a curvature that reduces the range of motion of a patient to an extent that inhibits normal bending. In such patients, the primary choice of treatment is Spinal Orthopedic Scoliosis Correction Surgery (SOSCS). SOSCS can improve a patient's appearance, along with the cardiopulmonary function, prevent further neurological deterioration, deformity progression, improve quality of life, and other general conditions that would prolong the patient's life expectancy.^[1]^ However, SOSCS is a complicated surgery and can result in spinal cord injury, such an injury can be caused as a result of misplacement of surgical instruments, or during procedures such as spinal canal decompression, osteotomy, or placement of spinal implants, where the procedures are performed in close proximity of the spinal cord.^[2,3]^ These injuries can cause serious complications such as paraplegia.^[4]^ To avoid such complications, intraoperative nerve assessment tools such as somatosensory evoked potentials and transcranial motor evoked potentials are commonly used to verify the integrity of the spinal cord. However, their reliability is questionable, and unlike the Stagnara Wake-up test^[5]^ remains the criterion standard procedure during SOSCS.^[6]^ Stagnara Wake-up test refers to waking up the patient during the surgery and commanding them to move their lower limbs to ensure the intactness of the spinal cord function (Table 1).

**Table 1:** Comparison of intraoperative blood oxygen saturation ($\bar{\text{x}}$ ± s, n = 20).

Timestamp^∗^	Group A	Group B	Group C	*P*
T-3	99.8 ± 0.41	100 ± 0	99.95 ± 0.22	.06
T-2	99.85 ± 0.37	100 ± 0	99.95 ± 0.22	.16
T-1	99.85 ± 0.37	100 ± 0	99.95 ± 0.22	.16
T0	99.85 ± 0.37	100 ± 0	99.95 ± 0.22	.16
T+1	99.85 ± 0.37	100 ± 0	99.95 ± 0.22	.16
T+2	99.85 ± 0.37	100 ± 0	99.95 ± 0.22	.16
T+3	99.85 ± 0.37	100 ± 0	99.95 ± 0.22	.16

∗ Timestamps defined in Table 2.

For an anesthesiologist, successful execution of a wake-up test requires that the patient wakes up quickly during the surgery, is able to understand and respond to the commands of the surgeon, and then goes back under and wakes up after the surgery with no memory of the test. For this reason, anesthesiologists need to carefully choose a combination of drugs that would allow for the smooth execution of the test. Although in recent years, intravenous anesthetics with fast clearance and short half-lives such as propofol and remifentanil have been widely used in intraoperative wake-up tests, there are issues related to delayed recovery, restlessness, pain, poor coordination, difficult hemodynamic stability, and other issues.^[7]^

Dexmedetomidine is a new type of α2 receptor agonist that acts by acting on the brainstem locus coeruleus nucleus, thereby inhibiting the release of norepinephrine which makes it a very effective sedative.^[8]^ In practice, it has proven to not only prevent delirium and anxiety, but also its sedative effect can make a patient transition easily between sleep and wakefulness.^[9]^ These characteristics make it an extremely useful drug that offers a safe way to sedate and wake-up patients during surgery as required while having few side effects. Many studies have proven that dexmedetomidine can be safely administered within the dosage range of 0.2 to 0.7 μg/(kg·h).^[10]^ However, previous studies employed American Society of Anesthesiologists (ASA) I-II patients which makes it difficult to recommend in ASA III patients. Several studies have shown that the application of dexmedetomidine while keeping it at maintenance dose during SOSCS is advantageous during intraoperative wake-up tests in ASA I-II patients; however, the use of dexmedetomidine in ASA III has not yet been reported in the literature.^[9]^

As the intraoperative wake-up test is such an important test in SOSCS and many of these patients can be ASA III, we found it necessary to test the safety of this drug on ASA III patients and to explore the feasibility of dexmedetomidine use in combination with propofol and remifentanil in critically ill patients (ASA III) undergoing intraoperative wake-up test during SOSCS and to compare the different doses of dexmedetomidine in such patients. Critically ill patients (ASA III) in our study had severe scoliosis with Cobb angle of >45 °, had a severe or greatly impaired pulmonary function with type 1 or type 2 respiratory failure, their cardiopulmonary function was at level 3, resulting in poor anesthetic tolerance and increased hemodynamic variability. We hypothesize that decreasing the dose of dexmedetomidine could lead to insufficient analgesic and sedative effects while larger doses may cause hypotension, sinus bradycardia or cardiac arrest, and so on.^[11]^ Therefore, we need to find an optimum dosage that would allow for sufficient analgesia, shorten wake-up times with dexmedetomidine, as well as minimum side-effects in ASA III patients. In accordance with previous studies on ASA II patients, 0.2 μg/(kg·h) had the least side effects while maintaining adequate anesthesia.^[12–14]^ Dosages up to 0.4 μg/(kg·h) have shown to be relatively safe to administer.^[15,16]^ Whereas dosage above 0.4 μg/(kg·h) were accompanied with increased side effects such as decreased motor evoked potentials.^[17,18]^ Furthermore, the critically ill patients the kind we included in this study, SOSCS was a long procedure that would result in an increased accumulative dosage. This could be challenging in terms of side effects or complications. Therefore, in the light of the results of previous studies along with the preliminary experimental results of this study, to ensure the safety of the patients, we decided to keep the maintenance doses relatively low, at 0.2, 0.3, and 0.4 μg/(kg·h) among groups A, B, and C, respectively. We would be able to determine whether these doses were safe to use among ASA III patients.

## 2 Method

Our study was a single-center, parallel, completely randomized, controlled, double-blinded clinical trial study with an allocation ratio of 1:1:1. It was retrospectively registered in the Chinese Clinical trial registry (registration no. ChiCTR2100049341, July 30, 2021) and was approved by the Ethics Committee of Chengdu Third People's Hospital (August 8, 2018) and informed consent from patients was obtained. The patients or their family members signed an informed consent form. A total of 78 patients who were required to undergo intraoperative wake-up test during SOSCS under total intravenous general anesthesia from January 1, 2020, to January 31, 2021, were assessed, patients aged ≤40 years with severe spinal deformity and ASA grade ≥grade III and other who met the inclusion criteria were included in this study. We excluded all patients with a history of neurological, psychiatric, or neuromuscular diseases; or those who were unable to accept wake-up tests due to language or hearing problems; patients with a history of long-term use of sedative drugs; patients with contraindications to dexmedetomidine; those who were too young to cooperate; or with prominent liver and kidney dysfunction; those who canceled the operation or voluntarily withdrew from the study before the anesthesia; the wake-up test was temporarily canceled during the operation; the anesthesiologist administered inhalation anesthetics during the operation, or the patients whose tracheal tube could not be removed after the operation were excluded from the study. We were left with 67 patients.

### 2.1 Sample size calculation and randomization

According to the pre-trail experimental results, the average wake-up time of group C was 21 minutes, and the average wake-up time of group B was 14 minutes, and the average standard deviation of wake-up time was 7 minutes. We set α = 0.05, 1-β = 0.85, and used the “power and sample size calculator” to calculate the sample size online, the total sample size was 54. Considering the loss to follow-up rate of 10%, it was estimated that we needed 60 cases to be included in our study. The subjects included in the study were randomly divided into 3 groups A, B, and C with a ratio of (1:1:1) with the help of computer software (SAS 9.1), giving each group 20 patients. We used the computer software to generate a random number which helped with the blinding and randomization of the study. Sixty sealed envelopes were made which had 20 of each of the 3 different dosages of dexmedetomidine, 0.2 μg/(kg·h), 0.3 μg/(kg·h), and 0.4 μg/(kg·h), the sealed envelopes were then sequentially numbered according to the computer-generated random number, and the nonresearchers who did not participate in the trial arranged the envelopes in the ascending order. Then when a patient was brought into the operating room for the said study, the researcher opened it and assigned the dosage on the infusion pump and wrote the random number of the patient's file, and replaced the envelope in a separate pile. If a patient was disqualified during or after the surgery, the nonresearchers would assign the same dosage to the next randomly assigned patient from the initial 67 patients assessed. This did not affect the randomization or the double blindness of the study; it was done to ensure an adequate sample size to be obtained. The anesthesiologists, patients, and surgeons were completely blinded from the group assignment of the patients.

### 2.2 Procedure

**Figure 1:**
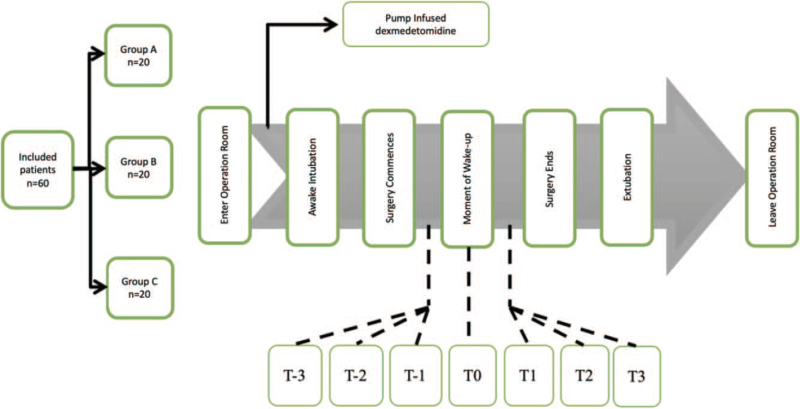
Procedure flowchart. Groups are defined in Method under sample size calculation and randomization, timestamps defined in Table 3.

We routinely monitored the basic vital signs and Bispectral Index (BIS) values of the patients after entering the room. All patients were intravenously administered phencyclidine hydrochloride 0.01 mg/kg (to inhibit salivary and airway glandular secretions, as intubation was done while patients were conscious) and methylprednisolone 40 mg. The procedure can be seen in Figure 1. Before induction of anesthesia, all patients were given a loading dose of 1 μg/kg/10 min of dexmedetomidine. The 3 groups were divided based on the dosage of dexmedetomidine they were administered. Group A, B, and C were each administered a continuous infusion of 0.2 μg/(kg·h), 0.3 μg/(kg·h), and 0.4 μg/(kg·h) of dexmedetomidine, respectively, until the end of the wake-up test. The Scoliosis Patients had a cephalopelvic ring stent implanted, which limited head movement and resulted in a difficult airway. For topical anesthesia, 1% tetracaine glue was applied to the oral surfaces, 3 mL of 2% lidocaine was injected into the tracheal walls with a thyrocricocentesis needle, an oxycodone injection of 0.1 mg/kg was given intravenously, and dacronine glue was administered to the tracheal cuff. Fiberoptic bronchoscopy was utilized to guide tracheal intubation under conscious sedation once the medication took effect.

After successful intubation, the patients were treated with midazolam 0.05 mg/kg, sufentanil 0.3 μg/kg, etomidate 0.2 mg/kg, and cisatracurium besylate. Cisatracurium besylate 0.2 mg/kg was used to induce anesthesia, and then the patients were connected to the breathing circuit of the anesthesia machine for mechanical ventilation and monitored for partial pressure of end-tidal carbon dioxide. After induction of anesthesia and intubation, Allen's test was performed to evaluate the condition of the radial artery, the arterial line was established in the left radial artery for invasive blood pressure measurement, and a triple-lumen catheter was placed in the right internal jugular vein to measure the central venous pressure (CVP), and bladder temperature was monitored. All patients were treated with propofol Target controlled infusion (TCI) 2 to 4 μg/mL, remifentanil TCI 2.5 to 4 ng/mL, and the corresponding maintenance dose of dexmedetomidine in each group. During the operation, the dosage of propofol and remifentanil was adjusted to maintain the BIS value between 40 and 60. The breathing parameters were adjusted, according to the need, to maintain the end-expiratory carbon dioxide partial pressure between 35∼45 mmHg; a surface heating blanket was used, liquid + blood products were brought up to body temperature before infusion, to maintain the patient's body temperature ≥36°C to eliminate the impact of change in body temperature on our research. Before the surgical incision, flurbiprofen axetil 50 mg, sufentanil 0.2ug/kg, and cisatracurium besylate 0.1 mg/kg was administered intravenously. Sufentanil and muscle relaxants were not administered later on. During the operation, the motor evoked potential (MEP) and somatosensory evoked potential (SSEP) were continuously monitored. Immediately after the completion of the wake-up test, the infusion of dexmedetomidine was stopped, whereas remifentanil and propofol (TCI concentration was adjusted according to BIS) infusion continued, and intravenous oxycodone injection 0.1 mg/kg and tropisetron 3 mg were administered. Propofol and remifentanil infusion was stopped after skin stitches were complete. After the operation, the tracheal tube was removed when the patient reached the extubation point.

### 2.3 Wake-up test procedure

During the operation, when the surgeon requested a wake-up test from time to time, drug infusion was halted among all 3 groups, except for the infusion of corresponding maintenance dose of dexmedetomidine in each group. The ventilator was used in the Synchronized Intermittent Mandatory Ventilation (SIMV) mode, combined with BIS, motor evoked potentials (MEP) and somatosensory evoked potentials (SSEP) monitoring results, when BIS >75, we called the patient's name every 20 seconds and commanded them to open their eyes and move their toes until they could respond to the instructions. The wake-up time and wake-up quality of the 3 groups of patients were recorded.

Successful Wake-up criteria: The patient responds and obeys the instructions when he hears the call.

Wake-up time: The time between discontinuing propofol and remifentanil until the patient hears the call and obeys the command.

Wake-up quality classification: We classified the responses of a patient to the commands into 4 grades (see Table 2).

**Table 2:** Wake-up quality classification.

Response level	Grade
The patient hears the call, opens eyes and is able to move toes as instructed.	I
The patient hears the call, opens eyes but is unable to immediately move toes as instructed.	II
The patient hears the call, opens eyes but is unable to move toes as instructed.	III
The patient hears the call, opens eyes but is unable to move toes as instructed and physical activity threatened the stability of internal fixation.	IV

#### 2.3.1 Observation landmarks

Primary outcome indicators:

1. Record and compare the wake-up time, wake-up quality, and adverse reactions during the wake-up period of the 3 groups of patients.

Secondary outcome indicators:

1. Follow-up with the patients on the first postoperative day, paying special attention to whether they have any memory of the wake-up test during the operation.
2. Retrospectively record and compare heart rate (HR), mean arterial pressure (MAP), blood oxygen saturation (SpO_2_), and BIS value of the 3 groups of patients at 7 timestamps over 30 minutes by collecting the full waveform and continuous data from the patient monitor. Each timestamp is divided by a time interval of 5 minutes starting at T-3 which is 15 minutes before the time of wake-up (T0) and ending at 15 minutes after wake-up T3.

Table 3 shows a list of the 7 timestamps with their respective definitions.

**Table 3:** Timestamps.

Timestamp	Definition
T-3	15 min Before wake-up
T-2	10 min Before wake-up
T-1	5 min Before wake-up
T0	Moment of wake-up
T1	5 min After wake-up
T2	10 min After wake-up
T3	15 min After wake-up

### 2.4 Statistical method

The mean ± standard deviation (x ± s) represents the measurement data conforming to the normal distribution, and the median (Md) and the interquartile range (P25–P75) represent the measurement data of the non-normal distribution. n (%) Represents a total number of patients, among which basic data such as the patient's age, height, arm length, anesthesia, and operation time, among others. We used the 1-way analysis of variance (ANOVA) or ANOVA rank-sum test. One-way variance was used to analyze the hemodynamic data, and Bonferroni was used to make pairwise comparisons among the 3 groups A, B, and C. *χ*^2^ Test was used to analyze n (%), and *P* < .05 indicated that the difference is statistically significant.

## 3 Results

**Figure 2:**
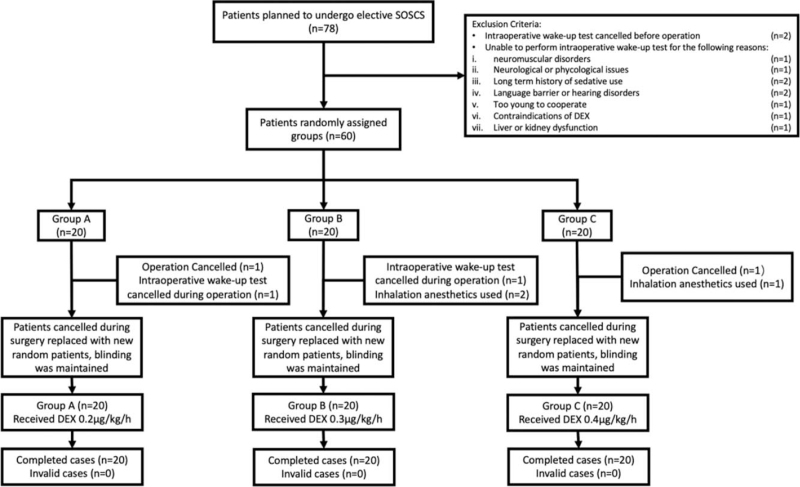
CONSORT flow diagram. DEX = dexmedetomidine, SOSCS = spinal orthopedic scoliosis correction surgery.

In this study, a single-center completely randomized double-blind method was used to enroll 60 patients who underwent SOSCS in the Department of Orthopedics, Chengdu Third People's Hospital from January 1, 2020 to January 31, 2021. Initially, we enrolled 78 patients but then excluded 18 for various reasons as shown in Figure 2.

### 3.1 Participant characteristics

There was no significant difference in age, gender, weight, height, arm length, body mass index (BMI), and ASA classification among the 3 groups of patients, as shown in Table 4.

**Table 4:** Participant characteristics ($\bar{\text{x}}$ ± s, n = 20).

Characteristics	Group A	Group B	Group C	*P*
Age, y	23.65 ± 8.37	24.80 ± 7.05	23.35 ± 7.99	.83
Sex, male (%)	10 (50%)	9 (45%)	9 (45%)	.94
Weight, kg	49.13 ± 11.05	45.68 ± 7.43	43.83 ± 10.64	.23
Height, cm	153.05 ± 9.69	147.15 ± 7.84	147.50 ± 13.07	.14
Arm length, cm	163.05 ± 7.66	161.40 ± 9.50	160.10 ± 9.46	.58
BMI, kg/m^2^	18.33 ± 3.51	17.67 ± 3.22	17.02 ± 3.37	.47
ASA Grade	III (100%)	III (100%)	III (100%)	1.00

ASA = American Society of Anesthesiologists, BMI = body mass index.

### 3.2 Comparison of indicators of wake-up time, wake-up quality, and incidence of adverse reactions during wake-up

Among the 3 groups of patients, there was a statistically significant difference in the wake-up time between group A and group C, as well as group B and group C (^∗^*P* = .02). There was no significant difference between the 3 groups in the quality of wake-up and the incidence of adverse reactions during the intraoperative wake-up test. As shown in Table 5, Table 6, and Table 7.

**Table 5:** Comparison of Wake-up Time ($\bar{\text{x}}$ ± s, n = 20).

	Group A	Group B	Group C	*P*
Wake-up time, min	14.95 ± 7.42^∗^	14.7 ± 6.52^∗^	21.3 ± 10.02	.02

**Table 6:** Comparison of wake-up quality (n%, n = 20).

Wake-up quality	Group A	Group B	Group C
I	19 (95)	19 (95)	16 (80)
II	1 (5)	1 (5)	4 (20)
III	0	0	0
IV	0	0	0

**Table 7:** Comparison of incidence of adverse reactions during the intraoperative wake-up test (n%, n = 20).

	Group A	Group B	Group C
Arrhythmia	0	0	0
Hypertension	1 (5)	0	1 (5)
Restlessness	0	0	1 (5)
Struggle	0	0	0

### 3.3 Comparison of intraoperative awareness

There was no significant difference in the incidence of intraoperative awareness among the 3 groups of patients. As shown in Table 8.

**Table 8:** Comparison of intraoperative awareness (n%, n = 20).

Intraoperative awareness	Group A	Group B	Group C
Yes	0	0	0

### 3.4 Comparison of intraoperative hemodynamic values

There was no significant difference in the heart rate between the 3 groups of patients at 7 times. However, there was a statistically significant difference in MAP at T-2 (∗*P* = .03) and T0 (∗*P* = .04) timestamps between group A and group B. The results are shown in Table 9 and Timestamps defined in Table 1.

**Table 9:** Comparison of intraoperative heart rate values ($\bar{\text{x}}$ ± s, n = 20).

Timestamp^∗^	Group A	Group B	Group C	*P*
T-3	76 ± 14.88	72 ± 14.96	76.25 ± 13.71	.59
T-2	75.05 ± 14.07	70.95 ± 16.24	76.85 ± 12.49	.42
T-1	76.3 ± 13.69	72.1 ± 16.81	77.75 ± 13.16	.45
T0	79.55 ± 15.61	76.15 ± 16.25	78.75 ± 13.81	.76
T+1	76.05 ± 16.39	75.35 ± 15.66	75.55 ± 11.34	.99
T+2	74.25 ± 15.06	76.5 ± 16.25	75.4 ± 11.51	.89
T+3	74.45 ± 16.51	76.7 ± 15.81	73.6 ± 12.34	.80

∗ Timestamps defined in Table 2.

**Figure 3:**
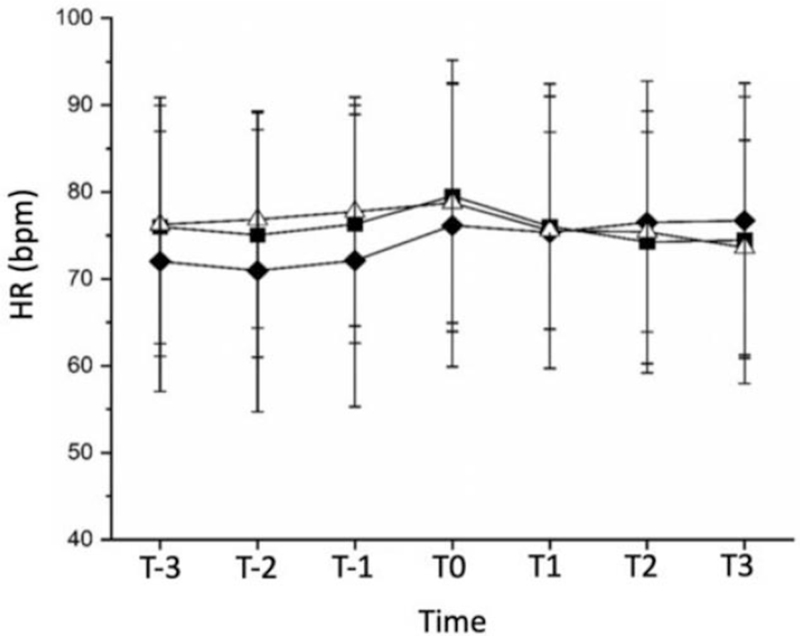
Heart rate variation trend chart. HR = heart rate, timestamps defined in Table 3. Groups are defined in Method under sample size calculation and randomization, ▪  = Group A, ♦ = Group B, Δ = Group C.

**Figure 4:**
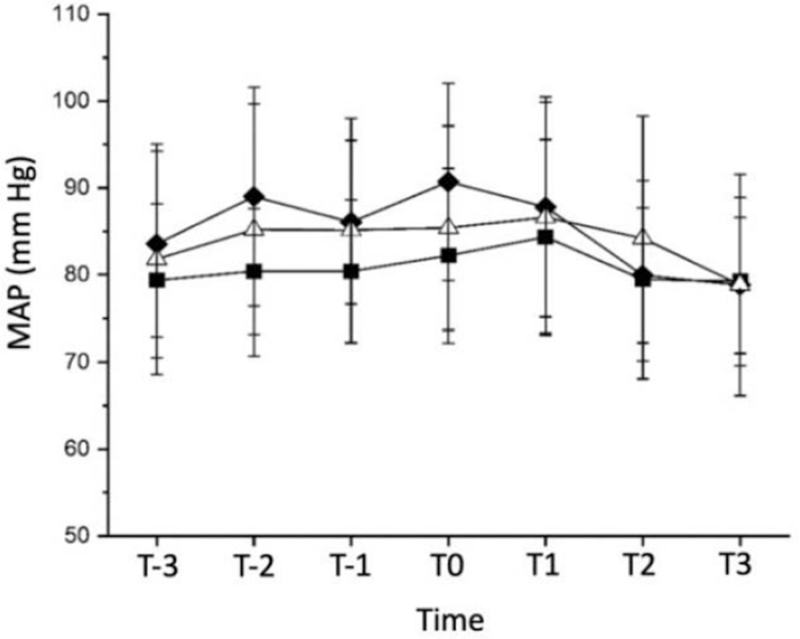
Mean arterial pressure variation trend chart. MAP = mean arterial pressure. Timestamps defined in Table 3. Groups are defined in Method under sample size calculation and randomization, ▪ = Group A, ♦ = Group B, Δ = Group C.

Table 10 and Figures 3 and 4 represent the data in the form of a graph.

**Table 10:** Comparison of intraoperative mean arterial pressure values ($\bar{\text{x}}$ ± s, n = 20).

Timestamp^∗^	Group A	Group B	Group C	*P*
T-3	79.35 ± 8.82	83.55 ± 10.67	81.8 ± 13.25	.49
T-2	80.4 ± 7.23	89 ± 12.58^∗^	85.2 ± 14.48	.08
T-1	80.4 ± 8.24	86.05 ± 9.42	85.15 ± 12.87	.19
T0	82.2 ± 10.04	90.7 ± 11.36^∗^	85.4 ± 11.73	.06
T+1	84.35 ± 11.22	87.8 ± 12.66	86.6 ± 13.27	.67
T+2	79.45 ± 11.39	79.95 ± 7.74	84.2 ± 14.07	.36
T+3	79.25 ± 9.64	78.8 ± 7.78	78.85 ± 12.70	.99

∗ Timestamps defined in Table 2.

### 3.5 Intraoperative hemodynamic variation (HR and MAP) trend

### 3.6 Comparison of intraoperative blood oxygen saturation

There was no significant difference in blood oxygen saturation (SpO_2_) among the 3 groups, as shown in Table 11.

**Table 11:** Comparison of intraoperative bispectral index values ($\bar{\text{x}}$ ± s, n = 20).

Timestamp^∗^	Group A	Group B	Group C	*P*
T-3	61.65 ± 3.51	61.5 ± 4.16	64.2 ± 4.54^∗^	.02
T-2	66.95 ± 5.44	67.15 ± 5.26	70.35 ± 2.11^∗^	.00
T-1	74.35 ± 3.41	76.05 ± 5.19	77.2 ± 2.40	.07
T0	85.85 ± 3.57	86.15 ± 4.11	87.15 ± 2.64^∗^	.01
T+1	69.4 ± 3.69	70.8 ± 3.99	71.3 ± 4.43	.31
T+2	64.25 ± 3.43	64.2 ± 2.59	64.15 ± 4.49	.99
T+3	59.75 ± 2.88	58.8 ± 3.71	57.45 ± 3.46	0.10

∗ Timestamps defined in Table 2.

### 3.7 Comparison of intraoperative bispectral index values

There was a statistically significant difference between the BIS values of group A and group C at 3 timestamps T-3 (∗*P* = .02), T-2 (∗*P* = .001), and T0 (∗*P* = .01), as shown in Table 12.

**Table 12:** Comparison of various indexes during operation ($\bar{\text{x}}$ ± s, n = 20).

	Group A	Group B	Group C	*P*
Anesthesia time at the beginning of intraoperative wake-up test, min	340.35 ± 75.58	379.70 ± 104.96	373.15 ± 93.99	.36
Total anesthesia time, min	475.50 ± 106.28	534.30 ± 112.62	510.65 ± 122.78	.27
Duration of Surgery, min	367.35 ± 112.05	432.55 ± 107.01	403.90 ± 99.894	.17
Blood loss volume, mL	1597.5 ± 1057.86	2132.5 ± 1547.52	1924.5 ± 1155.15	.41
Propofol dosage at the beginning of the intraoperative wake-up test, mg	1479 ± 329.46	1742.5 ± 409.88	1618 ± 360.93	.09
Total Propofol used, mg	1718 ± 364.17	2003.5 ± 393.17	1862 ± 417.32	.08
Remifentanil dosage at the beginning of intraoperative wake-up test, μg	2493 ± 421.49	2835 ± 543.01	2576 ± 550.54	.09
Total remifentanil used, μg	2746 ± 410.60	3043.5 ± 504.75	2852 ± 585.27	.18

### 3.8 Comparison of various indexes during operation

Differences in anesthesia time at the beginning of the intraoperative wake-up test, total anesthesia time, duration of surgery, blood loss volume, propofol dosage at the beginning of the intraoperative wake-up test, total propofol used, remifentanil dosage at the beginning of the intraoperative wake-up test, and total remifentanil used were compared among the 3 groups of patients and no statistical significance was found, as shown in Table 12.

## 4 Discussion

The incidence of adverse reactions during the wake-up period of the 3 groups of patients was low; the wake-up quality was at levels I and II ((Table 2) which is a good result. We also analyzed the hemodynamic indicators (HR, MAP) at 7 different time stamps which showed that there were statistically significant differences at T-2 (10 minutes before awakening) *P* = .03 and T0 (immediate awakening) *P* = .04 in MAP between group A and group B. The MAP fluctuations of group A at T-2 (10 minutes before awakening) and T0 (immediately awakening) were smaller. We are not able to explain the cause of these differences; it could be due to variations in the conditions of the patients or their extent of scoliosis, which might require further investigations. Regardless, the values are still well within the normal ranges; therefore, we believe they do not affect the results of our study. In terms of adverse reactions during the wake-up test, the incidence of hypertension in all 3 groups was lower. This is consistent with a previous study that showed that dexmedetomidine can provide hemodynamic stability due to its anti-sympathetic effect.^[19]^ Qingwu^[20]^ found that when dexmedetomidine was not used, and only propofol combined with remifentanil was used for intravenous anesthesia during SOSCS although the wake-up time was short, the incidence of agitation during the wake-up period was high, and the hemodynamic fluctuations during the wake-up tests were greater than that of propofol combined with sufentanil. However, the low incidence of agitation during the wake-up test in the 3 groups of patients in our study indicated that dexmedetomidine can significantly reduce the incidence of agitation due to its sedative effect, which is beneficial in critically ill patients.

### 4.1 Context to results

Our results show that there was no significant difference in the wake-up quality among the 3 groups and all other parameters that we observed were nominal, signifying that all 3 doses are safe to administer to ASA III patients. However, the purpose of our study was to find the optimum dosage which allows for the best sedative effects and decreased wake-up time. We found that among group A patients the wake-up time was 14.95 ± 7.42 minutes and 14.7 ± 6.52 minutes in group B. There was no significant difference in wake-up time between group A and group B. Both groups, however, were significantly shorter than group C in which the patients experienced longer wake-up times 21.3 ± 10.02 minutes (*P* = .02). We believe this is because of the side effects of dexmedetomidine as our patients were ASA III. As we know that dexmedetomidine has side effects that could cause cardiac arrhythmias, such as bradycardia, we assume that since our patients had a poorer cardiac function, it is possible that a higher dose of dexmedetomidine resulted in adverse effects on the hemodynamics which were although within the normal range but might have resulted in a prolonged wake-up time. Moreover, SOSCS is a very long surgery with a duration of averaging >6 hours, the accumulative dosage of dexmedetomidine can get much higher than in other surgeries with shorter durations, and for this reason for such critically ill patients undergoing SOSCS the side effects of dexmedetomidine might have become more pronounced resulting in prolonged wake-up times these results are consistent with the results of previous studies.^[21]^ Therefore, we suggest that 0.2 to 0.3 μg/(kg·h) as the optimum maintenance dose of dexmedetomidine can provide faster wake-ups while maintaining the wake-up quality. This is consistent with Chen et al^[22]^ indicating that the intraoperative low concentration of dexmedetomidine had fewer side effects and that continuous infusion of dexmedetomidine can also be successfully used for awakening critically ill patients during SOSCS.

However, we also find that it is safe to administer all 3 maintenance doses of dexmedetomidine during SOSCS that involves an intraoperative wake-up test in ASA III patients.

### 4.2 Limitations

This study was single-center research which means that the subjects were only from a particular area, although undefined, geographically it limits the subjects included. The sample size although calculated to be large enough is still quite small. Larger sample sizes might give more accurate results. Furthermore, the lowest dosage defined by this study is 0.2 μg/(kg·h) and the highest dosage as 0.4 μg/(kg·h), this study does not address dosages outside this range. Further studies are required to determine the safety of doses outside this range.

## 5 Conclusions

Doses of 0.2 to 0.3 μg/(kg·h) have shorter wake-up time and fewer hemodynamic fluctuations compared to 0.4 μg/(kg·h). However, all 3 doses of 0.2, 0.3, and 0.4 μg/(kg·h) of dexmedetomidine can be safely administered in critically ill (ASA III) patients with severe scoliosis undergoing intraoperative wake-up test during SOSCS.

## Author contributions

**Conceptualization:** Ting Yang, Muhammad Saqib Mudabbar.

**Data curation:** Ting Yang, Muhammad Saqib Mudabbar.

**Formal analysis:** Ting Yang, Muhammad Saqib Mudabbar, Hong Jia.

**Investigation:** Ting Yang, Muhammad Saqib Mudabbar.

**Methodology:** Ting Yang, Muhammad Saqib Mudabbar.

**Project administration:** Ting Yang.

**Resources:** Ting Yang.

**Software:** Ting Yang, Muhammad Saqib Mudabbar.

**Supervision:** Ting Yang, Tao Chen, Qiang Fu, Bin Liu.

**Validation:** Ting Yang, Muhammad Saqib Mudabbar, Hong Jia.

**Visualization:** Ting Yang, Muhammad Saqib Mudabbar.

**Writing – original draft:** Ting Yang, Muhammad Saqib Mudabbar.

**Writing – review & editing:** Ting Yang, Muhammad Saqib Mudabbar.
